# Regional myocardial contractility in Thalassemia Major by magnetic resonance tagging

**DOI:** 10.1186/1532-429X-16-S1-P244

**Published:** 2014-01-16

**Authors:** Antonella Meloni, Chiara Tudisca, Emanuele Grassedonio, Cristina Paci, Alessandra Quota, Petra Keilberg, Vincenzo Positano, Massimo Lombardi, Massimo Midiri, Alessia Pepe

**Affiliations:** 1 CMR Unit, Fondazione G. Monasterio CNR-Regione Toscana and Institute of Clinical Physiology, Pisa, Italy; 2 Istituto di Radiologia, Policlinico "Paolo Giaccone", Palermo, Italy; 3 Centro Trasfusionale, Ospedale S Maria alla Gruccia, Montevarchi, Italy; 4 Serv. Talassemia, Osp. "V. Emanuele III", Gela, Italy

## Background

Magnetic resonance (MR) tagging analyzed by dedicated tracking algorithms allows very precise measurements of myocardial motion and characterization of regional myocardial function. No extensive data are available in literature. Our aim was to quantitatively assess for the regional myocardial contractility in thalassemia major (TM) patients and to correlate it with heart iron overload and global biventricular function.

## Methods

Seventy-four TM patients (46 F; 31.8 ± 8.5 yrs) enrolled in the MIOT (Myocardial Iron Overload in Thalassemia) network underwent MR (1.5T). Three short-axis (basal, medial and apical) tagged MR images were analyzed off-line using harmonic phase (HARP) methods (Diagnosoft software) and the circumferential shortening (Ecc) was evaluated for all the 16 myocardial segments. Four main circumferential regions (anterior, septal, inferior, and lateral) were defined. The same axes were acquired by a T2* GRE multiecho technique to assess myocardial iron overload (MIO). Biventricular function parameters were quantitatively evaluated by cine images.

## Results

**Figure 1:**
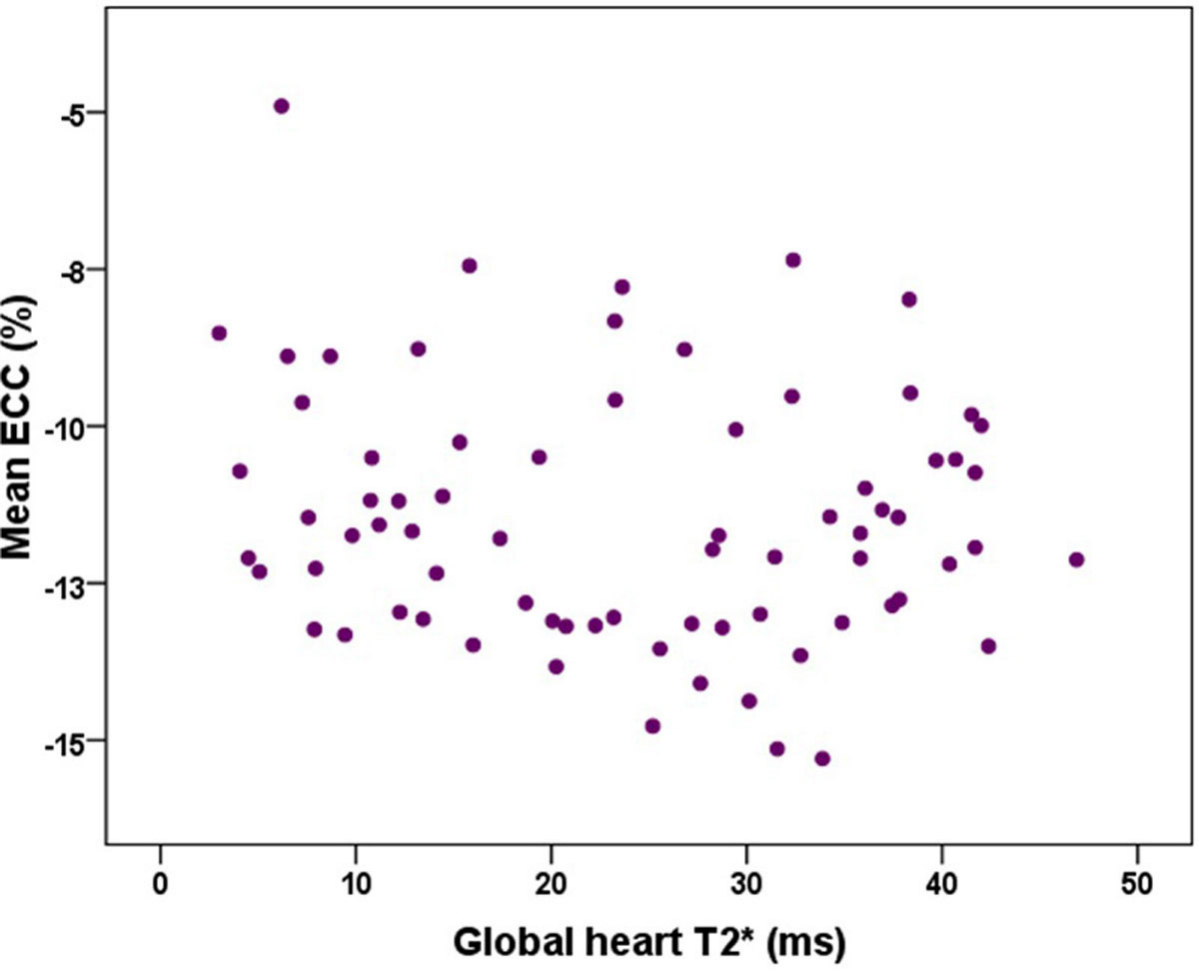
**Comparison with the study by Moore et al involving 31 healthy volunteers**.

Segmental ECC values ranged from -9.66 ± 4.17% (basal anteroseptal segment) to 13.36 ± 4.57% (mid-anterior segment). No significant circumferential variability was de-tected. Compared with previous studied healthy subjects, TM patients showed strain values sig-nificantly lower in all the circumferential regions at each level (mean difference from 4% to 13%; p < 0.001 for all the comparisons) (see Table 1). Segmental Ecc values were not significantly correlated with the correspondent T2* values and no correlation was detected considering the global values, averaged over all segmental values (see Figure 1). Three groups identified on the basis of cardiac iron distribution: no MIO, heterogenous MIO and homogeneous MIO. The global ECC was comparable among the three groups (-11.56 ± 1.60% vs -11.70 ± 2.43% vs -11.14 ± 1.95%; P = 0.602). Global ECC values were not significantly correlated with age and were comparable between the sexes. Circumferential shortening was not associated to left ventricular (LV) volumes and ejection fraction (with a p > 0.5 in all the comparisons).

**Table 1:** 

		Anterior	Septal	Inferior	Lateral
**Basal**	*Healthy*	-0.20 ± 0.03	-0.17 ± 0.03	-0.16 ± 0.03	-0.21 ± 0.03

	*TM*	-0.11 ± 0.04	-0.10 ± 0.03	-0.12 ± 0.04	-0.11 ± 0.03

		Diff = 0.09 p < 0.001	Diff = 0.07 p < 0.001	Diff = 0.04 p < 0.001	Diff = 0.10 p < 0.001

**Medium**	*Healthy*	-0.23 ± 0.04	-0.16 ± 0.03	-0.16 ± 0.05	-0.22 ± 0.03

	*TM*	-0.14 ± 0.05	-0.12 ± 0.03	-0.11 ± 0.04	-0.12 ± 0.03

		Diff = 0.09 p < 0.001	Diff = 0.04 p < 0.001	Diff = 0.05 p < 0.001	Diff = 0.10 p < 0.001

**Apical**	*Healthy*	-0.24 ± 0.06	-0.18 ± 0.03	-0.23 ± 0.04	-0.24 ± 0.04

	*TM*	-0.12 ± 0.04	-0.13 ± 0.04	-0.12 ± 0.05	-0.11 ± 0.04

		Diff = 0.12 p < 0.001	Diff = 0.05 p < 0.001	Diff = 0.11 p < 0.001	Diff = 0.13 p < 0.001

## Conclusions

TM patients showed a significantly lower cardiac contractility compared with healthy subjects, but this altered contractility was not related to cardiac iron, volumes and function.

## Funding

The MIOT project receives "no-profit support" from industrial sponsorships (Chiesi and ApoPharma Inc). This study was also supported by: "Ministero della Salute, fondi ex art. 12 D.Lgs. 502/92 e s.m.i., ricerca.

